# In Vivo and In Silico Assessment of Diabetes Ameliorating Potentiality and Safety Profile of *Gynura procumbens* Leaves

**DOI:** 10.1155/2022/9095504

**Published:** 2022-01-19

**Authors:** Md. Rafat Tahsin, Tanzia Islam Tithi, Sabiha Rahman Mim, Ehfazul Haque, Arifa Sultana, Nasiba Binte Bahar, Raju Ahmed, Jakir Ahmed Chowdhury, Abu Asad Chowdhury, Shaila Kabir, Fahima Aktar, Md. Sahab Uddin, Md. Shah Amran

**Affiliations:** ^1^Department of Pharmaceutical Sciences, North South University, Dhaka 1229, Bangladesh; ^2^Department of Pharmaceutical Technology, Faculty of Pharmacy, University of Dhaka, Dhaka 1000, Bangladesh; ^3^Department of Pharmaceutical Science, Uppsala University, Uppsala, Sweden; ^4^Molecular Pharmacology and Herbal Drug Research Laboratory, Department of Pharmaceutical Chemistry, Faculty of Pharmacy, University of Dhaka, Dhaka 1000, Bangladesh; ^5^Department of Pharmacy, Faculty of Pharmacy, University of Dhaka, Dhaka 1000, Bangladesh; ^6^Department of Pharmacy, Southeast University, Dhaka, Bangladesh; ^7^Pharmakon Neuroscience Research Network, Dhaka, Bangladesh

## Abstract

**Background:**

Diabetes mellitus is one of the most notable health dilemmas. Analyzing plants for new antidiabetic remedies has become an impressive territory for life science researchers. *Gynura procumbens* has long been used to treat diabetes. Thus, we strived to ascertain the hypoglycemic potentiality of extract of leaves of *G. procumbens* by in vivo and in silico approaches.

**Methods:**

Fresh leaves of *G. procumbens* were collected and shade-dried to prepare ethanolic extracts to evaluate pharmacological parameters. Diabetes was induced in rats via injecting alloxan through the intraperitoneal route at a dose of 150 mg/kg body weight. Humalyzer 3000 was used to perform a biochemical assay of collected samples from rats. Anti-hyperglycemic activity study along with overdose toxicity test was performed. The pharmacological activity of this plant was also evaluated through a molecular docking study. This in silico study investigated the binding affinity of natural ligands from *G. procumbens* against glycoside hydrolase enzymes.

**Results:**

We detected a peak plasma concentration of *G. procumbens* at 3 hours 45 minutes that is roughly similar to the peak plasma concentration of metformin. Again, in OGTT and anti-hyperglycemic tests, it has been ascertained that both plant extract and metformin can exert significant (*P* < 0.05) and highly significant (*P* < 0.01) hypoglycemic activity in a dose-dependent manner. Metformin exhibited better therapeutic efficacy than that of plant extract, but it possessed null statistical significance. Also, our safety profile expressed that, similar to metformin, the plant extract can restore the disturbed pathological state in a dose-oriented approach with a wide safety margin. In silico study also validated the potentialities of natural constituents of *G. procumbens. Conclusion*. This study suggested that G. *procumbens* can be considered as potential antidiabetic plant. Robust and meticulous investigation regarding plant chemistry and pharmacology in the future may bring about a new dimension that will aid in discovering antidiabetic drugs from this plant in the diabetes management system.

## 1. Introduction

Diabetes mellitus is characterized as one of the major epidemics in South Asia. Over 84 million people aged from 20 to 79 are suffering from type II diabetes that represents one-fifth of the total diabetic population [1, 2]. This number may get remarkably increased by 78% in 2045 [2]. Since the percentage of gross domestic product (GDP) expenditure in the health care sector has remained the same for the past two decades, this chronic disease leads to an enormous burden on individual household expenses and enhances diabetes-induced poverty. Hence, people choice shift towards plant-based treatment due to its abundance with lower price and very few side effects. Traditional medicine metformin belongs to the biguanides class and is widely used as first-line therapy for type II diabetes. Metformin exerts its action by inhibiting hepatic glucose production and decreasing intestinal glucose absorption [3]. There are other classes of drugs such as sulfonylureas and thiazolidinediones, GLP-1 receptor agonists [4] that act by stimulating insulin secretion and insulin sensitivity, respectively [5]. All these synthetic drugs are not excluded from causing mild to moderate side effects in patients, for example, weight loss or weight gain, nausea, gastrointestinal irritation, obesity, vitamin B12 deficiency, nocturnal hypoglycemia, and also contraindicated for the patient with renal dysfunction.

On the contrary, *G. procumbens* belonging to the family Asteraceae is commonly known as longevity spinach that grows broadly in India, Bangladesh, Southeast Asia, especially in Indonesia, Malaysia, and Thailand. Some of the traditional healing claims have been verified in scientific and pharmacological studies, including anti-herpes virus [6], anti-inflammatory [7, 8], anti-hyperlipidemic, anti-hyperglycemic [9–14], and anti-hypertensive activities [15–18]. Recently, particular attention has been given to *G. procumbens* in the pharmacology of antidiabetic medicinal plants perhaps because of its test data and efficiency in managing diabetes mellitus.

Algariri et al. and Hassan et al. have conducted their experimental studies on the rat model to show the mechanism of antidiabetic properties of *G. procumbens* [14, 19]. Both studies found the promising hypoglycemic efficacy of *G. procumbens* leaf extract that acts to decrease blood glucose levels in a similar way as to that of metformin. The phytochemical study of *G. procumbens* confirmed the presence of a significant amount of flavonoids and phenolic compounds that are essential for anti-hyperglycemic activity [19–22]. *G. procumbens* provides a large and safe therapeutic window with the lowest or no observable toxic effects. In the experiment, alloxan-induced diabetic rats were well-tolerated with the highest dose of *G. procumbens* extract. Hence, *G. procumbens* leaf extract could be used as a good alternative to traditional antidiabetic drugs. In addition, an in silico study is an important tool to determine the probable mechanism of action of natural constituents of *G. procumbens*. This study aims to compare the phytocompounds of *G. procumbens* against acarbose, which is an alpha-glucosidase and alpha-amylase inhibitor [23]. Docking of flavonoids and phenols as ligands with crystal targets alpha-glucosidase and alpha-amylase would define the binding affinity along with explaining the molecular pathway of inhibition by the extract of *G. procumbens* [24].

## 2. Materials and Methods

### 2.1. Drugs, Chemicals, and Instruments

Ethanol and alloxan were bought from Sigma Aldrich, Germany. Standard antidiabetic drug metformin was obtained from Healthcare Pharmaceutical Limited as a gift sample. The blood serum analyzing kits, total cholesterol, HDL, LDL, triglyceride, SGOT, SGPT, and creatinine were bought from Plasmatic Laboratory Product Ltd., UK. Humalyzer 3000 (semiautomated clinical chemistry analyzer originated from Medigroup Asia Limited, Cambodia) was used to assess the biochemical parameters, and the glucometer Alere GI of Alere Inc., USA, was bought from Shahbag, Dhaka, Bangladesh.

### 2.2. Plant Collection and Extract Preparation

*G. procumbens* leaves were collected from the medicinal plant garden of the Faculty of Pharmacy of the University of Dhaka. The authentication and taxonomic identification were then carried out. The plant specimen was kept at Bangladesh's National Herbarium according to their guidelines. The herbarium authorities assigned the accession number 47380, dated 11-2-2019 for future reference.

The leaves were shade-dried for 7–10 days before being coarsely pulverized. The powdered leaves were steeped in 70% ethanol for 96 hours while being vigorously shaken. The extract was filtered when it had finished soaking, and the filtered liquid was collected. The extracted solution was then transferred to a rotary evaporator machine to concentrate it. Finally, the dried extract was carefully collected and stored for future use.

### 2.3. Experimental Animal Handling

Adult healthy male Wistar rats (125–200 gm) were collected from the Zoology Department of Chittagong University, Chittagong, Bangladesh, and kept at the Institute of Nutrition and Food Science, University of Dhaka, under a 12 hour dark/light cycle at a constant temperature of 25°C. Standard pellet diet and clean water were provided regularly. The rats were kept there for acclimatization before the investigation started. All experimental procedures involving rats were carried out in compliance with the guidelines established by the Institutional Animal Ethics Committee (IEAC). Animals were treated and handled in accordance with the guidelines of the Swiss Academy of Medical Sciences (SAMS) and the Swiss Academy of Sciences (SCNAT).

### 2.4. Experimental Guidelines

All tests were carried out in accordance with the ethical principles outlined in the Declaration of Helsinki 2013. The entire course of this research was performed in strict adherence with the “3R” principles, a fundamental element of Swiss and international guidelines regulating the exploitation of animals for experimental purposes. The first “R” stands for “replacement” that involves both absolute replacements (replacing animal models with computer-simulated models) and relative replacements (replacing live animals by cell or tissue cultures, or replacing vertebrates with invertebrates). In order to adhere to the concept of “replacement,” our study was initiated with an in silico analysis. However, this model failed to generate adequate data. Hence, an animal model was employed for further investigation. Since investigation for antidiabetic potential necessitates animals with specified pancreas and beta cells, mammalian vertebrates (i.e., rats) were chosen instead of invertebrates. The second “R” stands for “reduction,” which refers to any approach that will lead to fewer animals being utilized to achieve sufficient data to solve the research queries or in optimizing the information acquired from each animal. To comply with this guideline, the sample size was estimated using the “power analysis method,” and on the basis of this calculation, ten rats per group were taken for this study. The third “R” denotes “refinement,” which implies lessening the amount of suffering caused to the experimental animals by alleviating their pain. For this purpose, the tail tips of rats were rubbed with isopropyl alcohol before and after each measurement of blood glucose levels to make the procedure more tolerable and lessen the pain caused by pinching. The rats were well-fed throughout this entire research, and at the end of this study, they were euthanized in a painless manner under the influence of general anesthesia according to the 2013 edition of the Guidelines for the Euthanasia of Animals.

### 2.5. Sample Size Estimation for the Animal Model

The sample size of our animal model study was determined applying the “power analysis method.” Calculations were performed manually using the following formula:

(1) $$\begin{array}{c} \text{sample size}=2\;{\text{SD}}^{2}\frac{{\left(\left(\text{Z}\alpha /2\right)+\text{Z}\beta \right)}^{2}}{d^{2}}, \end{array}$$

where SD = standard deviation from pilot study, *Zα*/2 = *Z* 0.05/2 = *Z* 0.025 = 1.96 (according to *Z* table) with 5% type 1 error rate, *Zβ* = *Z* 0.20 = 0.842 (according to *Z* table) at 80% power. *d* = difference between two (pretreatment and posttreatment) mean values of blood glucose levels = effect size.

The theoretical sample size was adjusted for the expected attrition of the rats. From our prior experience, it was observed that alloxan treatment might result in the death of 10% of rats. So the actual sample size was estimated by dividing the theoretical sample size by 0.9.

In our pilot study, ten rats were employed as “alloxan control,” and on the basis of the preceding calculation, each of our preclinical trials included ten rats. Following intraperitoneal administration of alloxan at a dose of 150 mg/kg body weight, the rats were left untreated for about four weeks. This resulted in a substantial increase in their blood glucose levels. After four weeks, the mean blood glucose level of rats was found to be 24.74 mmol/L, with a standard deviation (SD) of 5.12.

According to our prior experiment, it can be interpreted that if the mean value of blood glucose is found to be 18.05 mmol/L following treatment with the plant extract, it can be inferred that the extract can significantly lower the rise in blood glucose level (*p* < 0.05).

So, *d* = difference between two mean values = 24.74 – 18.05 = 6.85.

Hence, theoretical sample size = 2 SD^2^ (Z*α*/2 + Z*β*)^2^/d^2^ = 2 × (5.12)^2^ × (1.96 + 0.842)^2^/(6.85)^2^ = 8.77.

Then we adjusted this theoretical sample size for expected attrition to obtain the actual sample size.

So actual sample size = 8.77/0.9 = 9.74.

Therefore, ten rats per group were taken for the study.

During this COVID-19 induced lockdown, the rats were kept in the animal house, and the lab curator was the sole person assigned with the responsibility of caring for these animals. We, the researchers, were allowed to visit the lab only twice a week. Apart from this pandemic period, we used to observe the rats on a daily basis throughout their maintenance time. Most likely, some factors regarding the pandemic-related difficulties can have an effect on the increase in sample size. Based on our calculations, a minimum of ten rats were required in each group in order to increase the validity of our research findings.

### 2.6. Dose Selection

A pilot analysis was performed prior to the commencement of the study. From this pilot investigation, it was noted that the plant extract (*G. procumbens*) began exerting its pharmacological effect at a dose of 500 mg/kg, denoting its MEC (minimum effective concentration) value at a greater dose than 500 mg/kg. As the dose was increased, a constant rise in this effect was observed. Eventually, when the dose was raised from 1,000 mg/kg to 1,200 mg/kg, no noticeable effect on the pharmacological action was detected. This indicated that the receptors related to the pharmacological action of the plant began saturated at a dose of 1,000 mg/kg. The doses of Metformin (standard drug) were chosen in the same way.

### 2.7. Biological Sample Collection

Blood samples were collected via puncturing the tip of the tail of rats for the assessment of blood glucose levels. On the contrary, blood was collected by puncturing the heart immediately after sacrifice and taken to a microcentrifuge tube. The collected samples were centrifuged at 5,000 rpm for 5 minutes to obtain the supernatant fluid. This fluid was then transferred to another microcentrifuge tube to conduct biochemical assays.

The kidney and liver were immediately separated from the animal body after sacrifice and washed thoroughly by ice-cold saline in order to conduct kidney and liver function tests.

### 2.8. Experimental Design

The body weight of individual rats was weighed, and animals were divided into different groups to check peak plasma concentration (Table 1), oral glucose tolerance test (Table 2), anti-hyperglycemic activity analysis (Table 3), and overdose toxicity analysis (Table 4) where an even distribution of rodents as per their body weight has been taken place, and each group contained ten rats.

In Table 3, the alloxan control group represents rats that were treated with alloxan only. N/A refers that no therapeutic treatment was administered to rats belonging to this group.

### 2.9. Evaluation of Antidiabetic Properties

Alloxan was applied to induce diabetes in the rat model. Alloxan was first dissolved in a cold citrate buffer (0.1 M; pH = 4.5). Then the rats were treated with this alloxan at a dose of 150 mg/kg body weight through their intraperitoneal routes. After alloxan administration, they were monitored for hyperglycemia by measuring blood glucose levels four times a day at a six-hour interval, and within 72 hours post alloxan therapy, it was found that all alloxan-treated rats had an average blood glucose level greater than 15 mmol/L that clearly indicated their hyperglycemic or diabetic conditions. Metformin and extract of *G. procumbens* treatments were administered to rats via the oral route.

### 2.10. Enzymatic Activity Assay

The activities of hexokinase, aldolase, phosphoglucoisomerase, fructose-1, 6-diphosphatase, and glucose-6-phosphatase were analyzed from the liver and kidney [25–29]. Microdissection methods have been used to conduct various sensitive enzymatic assays on renal and hepatic functions for examining the cellular distribution of different enzymes on different metabolic pathways [30–32]. Particularly, enzymes that catalyze irreversible reactions in gluconeogenesis are mostly available in the proximal tubular region in the kidney [31, 33] along with the periportal region of liver acinus [34–36], whereas enzymes associated with glucose degradation are mostly available in the distal regions of the nephron [37–39] as well as centrilobular hepatocytes [35, 40–43].

### 2.11. Estimation of Biochemical Parameters

Blood glucose level was assessed using a glucometer. Besides making use of Humaluzer 3000, lipid profile, kidney, and liver functioning tests were performed. Furthermore, the activities of gluconeogenic and glycolytic enzymes were analyzed from the kidney and liver samples.

### 2.12. Overdose Toxicity Determination

To determine the toxicity of an overdose, 50 rats were separated into five groups. An overdose is defined as a dose containing 50 times the medium dose.

The actual purpose of the overdose toxicity study was to determine the minimum dose of any of the treatments (either metformin or *G. procumbens*) that would be lethal for 100% of rats. For this study, we took 50 rats and split them into five classes to study the effects of overdosing. Rats in groups 2 and 4 were treated with metformin, whereas rats in groups 3 and 5 were treated with *G. procumbens* in increasing dose levels (about 5 times, 10 times, 20 times, 30 times, 40 times, and finally 50 times) of the medium dose. The rats were kept under continuous observation until 100% lethality was noticed in any of the treatment groups (either metformin-treated or *G. procumbens*–treated groups). At a dose 50 times higher than the medium dose, all metformin-treated rats (groups 2 and 4) were found dead, whereas *G. procumbens*–treated rats (groups 3 and 5) remained alive. Hence, we considered this dose (50 times the medium dose) as an overdose. Our toxicity trial was terminated at this point as 100% lethality was attained in one of the treatment groups (i.e., metformin-treated groups).

### 2.13. Docking Studies of *Gynura procumbens* Phytochemicals to Characterize Different Pharmacological Activities

After an extensive literature study, it was found that the *G. procumbens* extract produced an antidiabetic effect through the inhibition of alpha-amylase and alpha-glucosidase enzymes. In order to perform in silico molecular docking study against these two endogenous peptides, a list of 90 compounds of different chemical classes was prepared through a meticulous literature review. The 3D structures of these compounds were downloaded from PubChem and later optimized through PyRx. Crystal structure of alpha-amylase (3BAJ) and N-terminal alpha-glucosidase (maltase-glucoamylase) (2QMJ) were downloaded from the RCSB-PDB. This server was also used to determine the binding site of the standard acarbose, which was later specified in the docking process. These macromolecules were prepared for docking study using PyMol. Next, molecular docking was performed using Autodock Vina, and the results were sorted using Microsoft Excel. Moreover, an interaction study was conducted using the Biovia Discovery Studio Visualizer.

### 2.14. Statistical Analysis

All study parameters belonging to each group displayed as mean ± SD. The “one-way ANOVA test” was conducted to interpret intergroup heterogeneity in terms of diverse biological parameters to determine the statistical significance. “SPSS 16” software was used for the analysis. The result was considered statistically significant when “*p*” value was obtained less than 0.05 (*p* < 0.05) and highly significant when the “*p*” value was found less than 0.01 (*p* < 0.01).

## 3. Results

### 3.1. Determination of Peak Plasma Concentration

**Figure 1:**
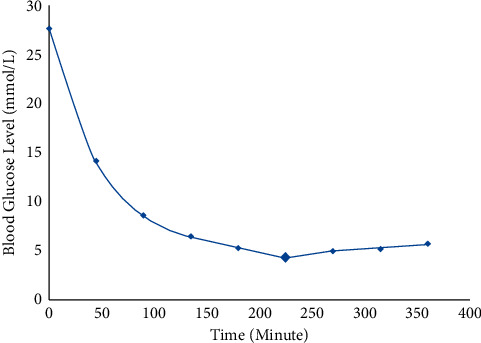
Peak plasma concentration of blood glucose level after administration of the extract of *G procumbens* in rats.

In this study, ten alloxan-induced rats were given a 2 g/kg dose of *G. procumbens* as shown in Table 1. After that, blood glucose levels were tested every 45 minutes.  Figure 1 shows that blood glucose levels are dropping before they reached 225 minutes, or 3 hours and 45 minutes. As a result, the peak plasma concentration may exist between 3 and 4 hours, and the peak plasma time was obtained at 3 hours 45 minutes when the concentration could be found maximum.

### 3.2. Impact on Body Weight during Treatment

**Figure 2:**
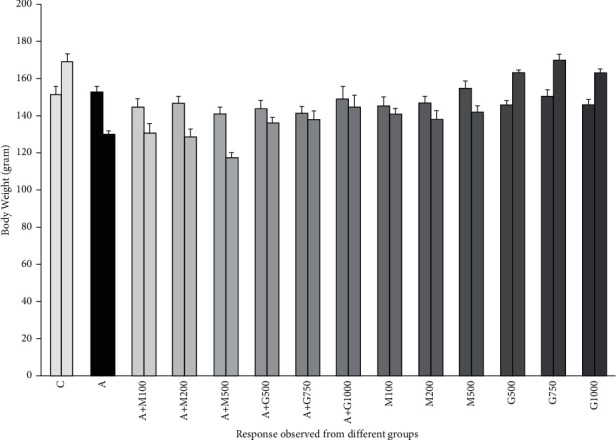
Body weight of rats before the initiation and after the termination of the experiment. Values were expressed as mean ± SD (C = control group, A = alloxan-induced group, M = metformin, A + M = alloxan + metformin, A + *G* = alloxan + *Gynura*, and *G* = *G. procumbens*).

In our experiment, alloxan significantly reduced the body weight of experimental rodents. In the treatment groups, plant extract successfully elevated the body weight of alloxan-induced diabetic rats, but metformin played a role in further reducing the body weight as shown in Figure 2.

### 3.3. Oral Glucose Tolerance Test

**Figure 3:**
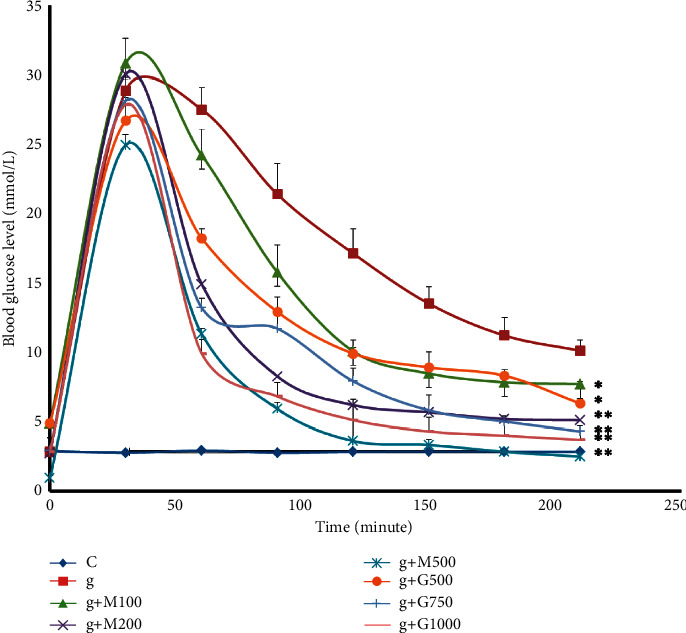
Blood glucose level of rats belonging to eight groups throughout receiving respective treatments. Values were expressed as mean ± SD (*n* = 10/group). *∗p* < 0.05 and *∗∗p* < 0.01 indicate significant differences from the disease group. For better visualization, 2 was added to the value of blood glucose level of low dose and 2 was subtracted from the value of the blood glucose level of high dose (C = control group, g = glucose-treated group, M = metformin, and *G* = *G. procumbens*).

We divided 80 rats into 8 classes for the oral glucose tolerance test. For OGTT, the rats were kept on an overnight fast prior to glucose administration. Except for the rats in group 1, all of the rats were induced with 10 g/kg glucose, and the blood glucose levels were tested after 30 minutes, and then all of the rats in groups 3–8 were induced with the respective treatment. In this test, blood glucose levels for all rats were checked for each 30 minutes interval for 3.5 hours.  Figure 3 shows that the extract can significantly reduce the elevated blood glucose level of nondiabetic glucose-induced rats.

### 3.4. Change in Blood Glucose Level

We took 140 rats and divided them into 14 classes to see how the extract affected blood glucose levels. Initially, blood glucose levels were determined, and alloxan was injected into rats in groups 2–8. Within 3 days, diabetes had been induced. We kept them untreated for 14 days. Then, on day 14, the treatment began. Rats in groups 3–8 were given their respective medications and doses until day 42. As a result, rats in groups 9–14 were also given their medications, but these groups were not treated with alloxan. From days 14 to 42, they were given medication. In addition, rats in group 1 were given standard food and water.

**Figure 4:**
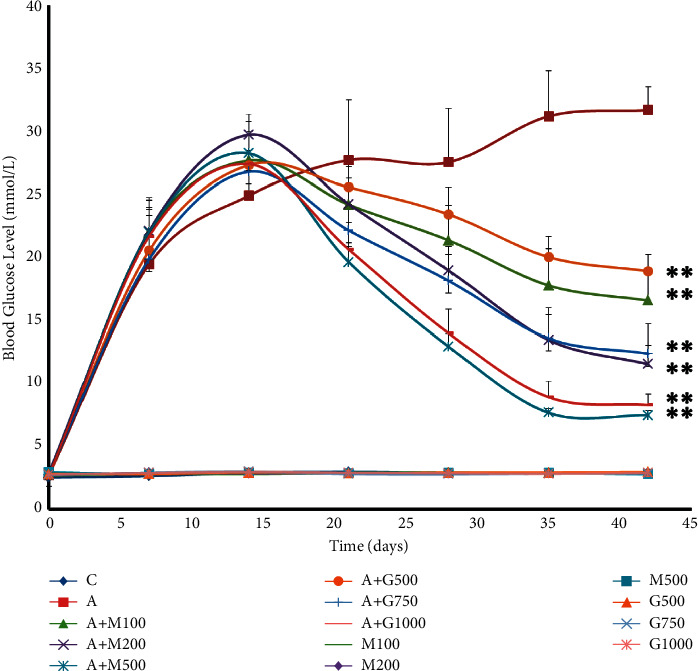
Blood glucose level of rats belonging to 14 groups throughout receiving respective treatments. Values were expressed as mean ± SD (*n* = 10/group). *∗p* < 0.05 and *∗∗p* < 0.01 indicate significant difference from the disease group (C = control group, A = alloxan-treated group, M = metformin, A + M = alloxan + metformin, A + *G* = alloxan + *Gynura*, and *G* = *G. procumbens*).

Alloxan is one of the most available compounds for inducing diabetes in experimental rodents. According to our study, the blood glucose levels of group 1 rats were detected to be normal that is shown in Figure 4. However, in the diabetic controlled group, due to destruction of beta cells and untreated condition, the blood glucose level was higher than all other groups. In all the metformin and extract-treated groups, the elevated blood glucose levels were decreased in the same pattern but not to the same extent. Nevertheless, the reduction of blood glucose in the metformin-treated group was a little bit higher than that of the extract-treated group, but it does not possess any statistical significance.

### 3.5. Kidney Function Test

**Figure 5:**
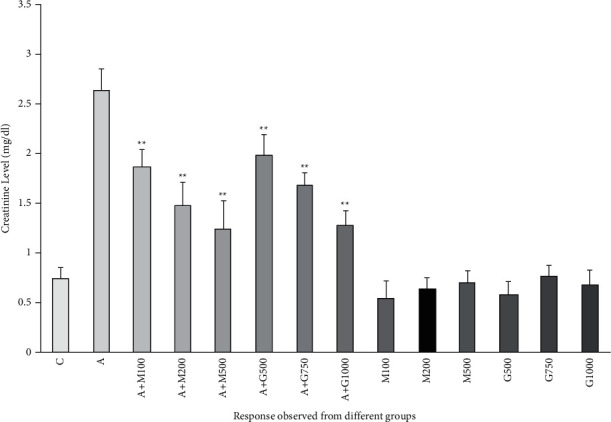
Creatinine level of rats belonging to 14 groups throughout receiving respective treatments. Values were expressed as mean ± SD (*n* = 10/group). ^∗^*p* < 0.05 and ^∗∗^*p* < 0.01 indicate significant difference from the disease group (C = control group, A = alloxan-treated group, M = metformin, A + M = alloxan + metformin, A + *G* = alloxan + *Gynura*, and *G* = *G. procumbens*).

In our study, creatinine level was obtained to be lower in the treatment groups in contrast with the alloxan control group as shown in Figure 5. The highest level of creatinine was observed in the alloxan control group due to its destructive effects. No significant difference in creatinine deduction was found between metformin and extract-induced treatment groups.

### 3.6. Liver Function Test

**Figure 6:**
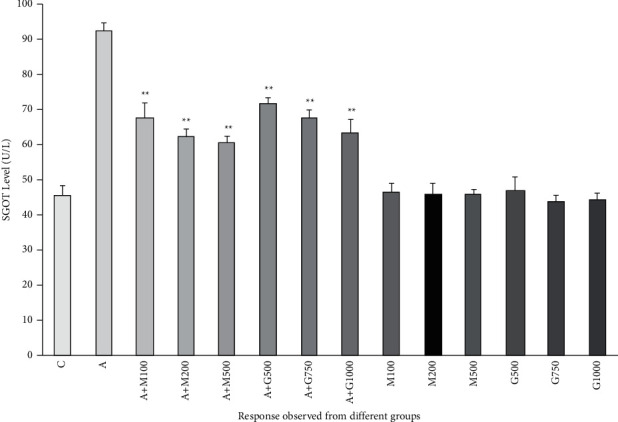
SGOT level of rats belonging to 14 groups throughout receiving respective treatments. Values were expressed as mean ± SD (*n* = 10/group). ^∗^*p* < 0.05 and ^∗∗^*p* < 0.01 indicate significant difference from the disease group (C = control, A = alloxan, M = metformin, A + M = alloxan + metformin, A + *G* = alloxan + *Gynura*, and *G* = *G. procumbens*).

**Figure 7:**
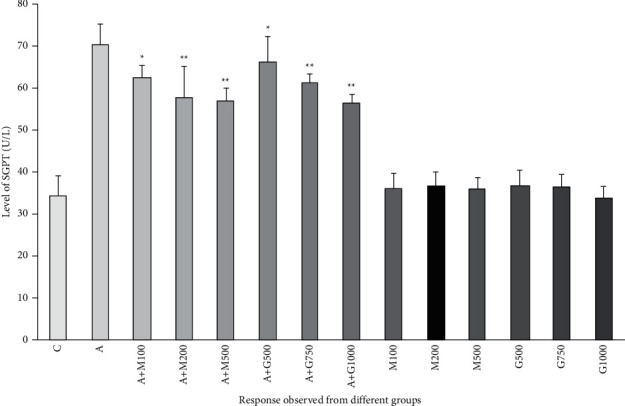
SGPT level of rats belonged to 14 groups throughout receiving respective treatments. Values were expressed as mean ± SD (*n* = 10/group). ^∗^*p* < 0.05 and ^∗∗^*p* < 0.01 indicate significant difference from the disease group (C = control group, A = alloxan-treated group, M = metformin, A + M = alloxan + metformin, A + *G* = alloxan + *Gynura*, and *G* = *G. procumbens*).

In the liver functioning test, the SGOT and SGPT levels were also enhanced significantly in alloxan-induced diabetic rats. The SGOT and SGPT levels of treatment groups were lower than all the other groups, including the alloxan control group. In the treatment groups containing the extract, the protective effect of *G. procumbens* extract declined the liver enzymes. It reduced the abnormally increased level of SGOT and SGPT levels as shown in Figures 6 and 7.

### 3.7. Lipid Profile Function Test

**Figure 8:**
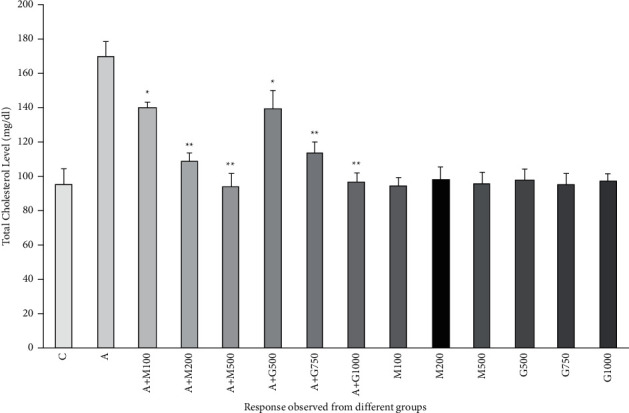
Total cholesterol level of rats belonging to 14 groups throughout receiving respective treatments. Values were expressed as mean ± SD (*n* = 10/group). ^∗^*p* < 0.05 and ^∗∗^*p* < 0.01 indicate significant difference from the disease group (C = control group, g = glucose-treated group, M = metformin, A + M = alloxan + metformin, A + *G* = alloxan + *Gynura*, and *G* = *G. procumbens*).

**Figure 9:**
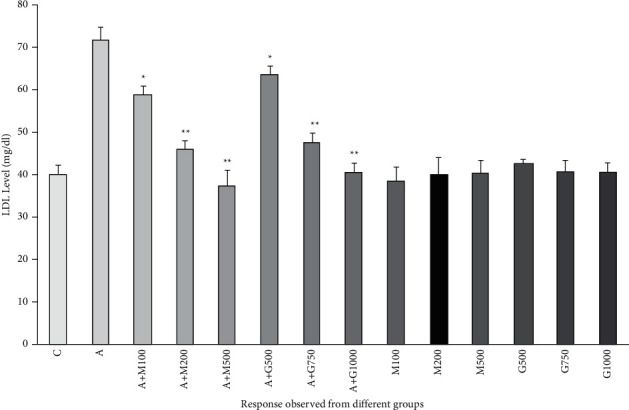
LDL level of rats belonging to 14 groups throughout receiving respective treatments. Values were expressed as mean ± SD (*n* = 10/group). ^∗^*p* < 0.05 and ^∗∗^*p* < 0.01 indicate significant difference from the disease group (C = control group, A = alloxan-treated group, M = metformin, A + M = alloxan + metformin, A + *G* = alloxan + *Gynura*, and *G* = *G. procumbens*).

**Figure 10:**
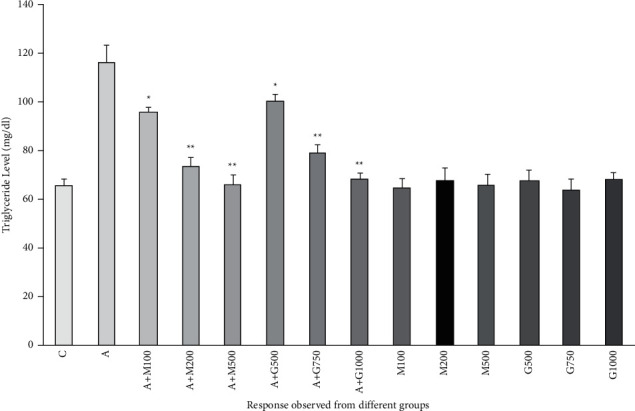
Triglyceride level of rats belonging to 14 groups throughout receiving respective treatments. Values were expressed as mean ± SD (*n* = 10/group). ^∗^*p* < 0.05 and ^∗∗^*p* < 0.01 express significant difference (*p* < 0.05) and high significant difference (*p* < 0.01) from the disease group, respectively (C = control group, A = alloxan-treated group, M = metformin, A + M = alloxan + metformin, A + *G* = alloxan + *Gynura*, and *G* = *G. proc*umbens).

**Figure 11:**
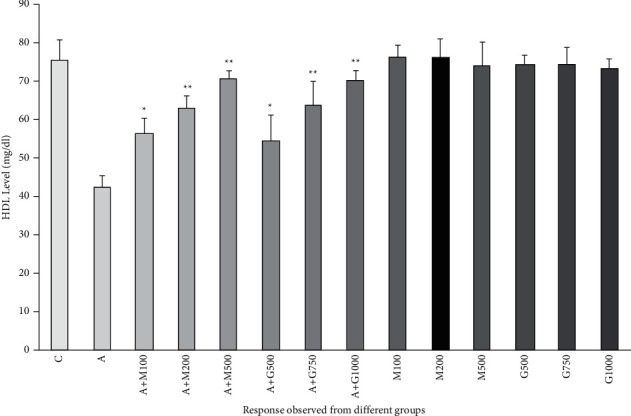
HDL level of rats belonging to 14 groups throughout receiving respective treatments. Values were expressed as mean ± SD (*n* = 10/group). ^∗^*p* < 0.05 and ^∗∗^*p* < 0.01 indicate significant difference from the disease group (C = control group, A = alloxan-treated group, M = metformin, A + M = alloxan + metformin, A + G = alloxan + *Gynura*, and *G* = *G. procumbens*).

Total cholesterol, LDL, and triglyceride levels of treatment groups were lower than alloxan-controlled groups as alloxan also increased the level of cholesterol, LDL, and triglyceride level as shown in Figures 8, 9, and 10, respectively. HDL level was also found to be highest in the negative control group and the lowest in the alloxan control group as shown in Figure 11. As a consequence, the level of HDL was increasing with the enhancement of dose both in the case of metformin and plant extract.

### 3.8. Glycolytic and Gluconeogenic Enzyme Activity Test

The activities of glycolytic enzymes were found abnormal in diabetic rats. In contrast, the treatment groups reflected the effectivity of plant extract and metformin by reversing that condition to a normal level as presented in Table 5 and 6. The hexokinase and phosphoglucoisomerase activities were seen to be decreased, and aldolase activity was seen to be increased in the alloxan control group compared to the treatment groups. *G. procumbens* extract significantly normalized the activities of glycolytic enzyme in a dose-dependent manner. A similar effect was obtained in the case of *Terminalia arjuna* extract [52].

In terms of gluconeogenic enzymes, the activities of fructose-1, 6-diphosphatase, and glucose-6-phosphatase (kidney and liver) were found to be elevated in the alloxan control group as shown in Table 7 and 8. After administering metformin and *G. procumbens* extract orally, the condition was reversed in a dose-dependent manner. A similar effect was obtained in the case of *T. arjuna* extract [44].

### 3.9. Overdose Toxicity Study

Rats from both the normal and the alloxan-induced metformin-treated groups died in this experiment. All of the rats in the alloxan-treated group died within 3 hours, while the average rats in the metformin-treated group died within 4 hours. However, both classes of rats treated with alloxan + *G. procumbens* and *G. procumbens* remained alive.

**Figure 12:**
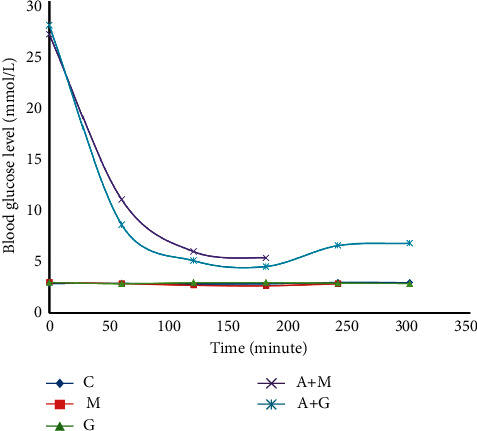
Blood sugar level of rats belonging to 1 to 5 groups throughout receiving respective treatments (C = control group, M = metformin, A + M = alloxan + metformin, A + G = alloxan + *Gynura*, and *G* = *G. procumbens*).

In an overdose toxicity study, it has been observed that even 50 times greater than that of the medium dose (750 mg/kg) of *G. procumbens* did not cause lethality in rodents, indicating the broad safety profile of *G. procumbens*. On the contrary, all metformin-treated rats (i.e., 100% rats) died within three hours of receiving a 50-fold higher dose of the drug as illustrated in Figure 12.

### 3.10. Docking Studies

**Figure 13:**
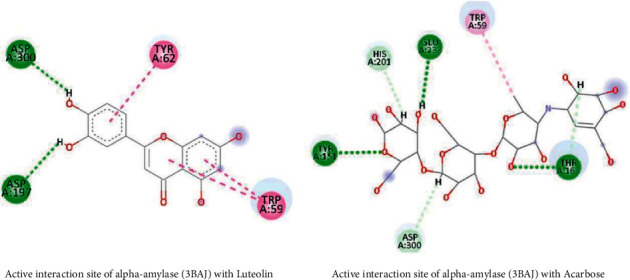
Active site interaction between alpha-amylase complex with the flavonoid and reference compound.

**Figure 14:**
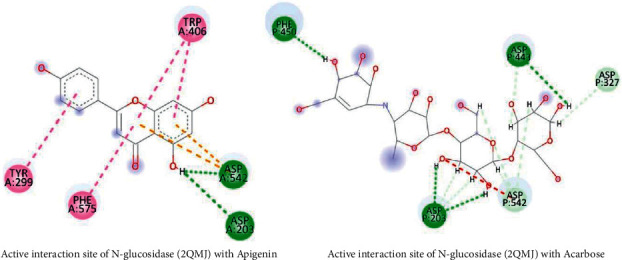
Active site interaction between N-glucosidase complex with the flavonoid and reference compound.

The results of the docking study against 3BAJ and 2QMJ are presented in Table 9 and 10, respectively. It is found that a total of 22 compounds displayed better binding as compared to the standard acarbose in the case of alpha-amylase. Twenty compounds also found stronger binding affinity compared to acarbose in the case of the N terminal maltase-glucoamylase. In both docking studies, flavonoids compounds showed higher binding affinity and similar interaction compared with reference drug, acarbose, as shown in Figures 13 and 14.

## 4. Discussion

Alloxan-induced diabetic groups had lower body weight than normal, and plant extract was observed to reverse this condition in a dose-dependent manner [45]. Apart from that, it was obtained that the extract of *G. procumbens* can effectively reduce the elevated blood glucose level gradually in alloxan-induced diabetic rats. Prior studies also supported this data [19]. Alloxan devastatingly increased the creatinine level in rats whereas *G. procumbens* reversed the condition [46, 47].

In the case of blood glucose-lowering effect, SGOT, SGPT, creatinine, total cholesterol, LDL, triglyceride, and HDL levels of all the drug-treated and extract-treated groups showed better conditions than the diabetic control group but worsened the condition than the healthy control group and among the drug and extract-treated groups, the drug-treated groups showed a little bit better results in all these parameters; still, no statistical significance (*P* > 0.05) was found when a comparison was drawn between these two. The condition was found to improve with the enhancement of the dose. The treatment of *G. procumbens* extract reversed the abnormal level of lipid profile towards the normal state in previous studies [48, 49]. Additionally, we observed that the SGOT, SGPT, creatinine, total cholesterol, HDL, triglyceride, and blood glucose levels of normal healthy rat treated with *G. procumbens* and metformin was almost similar to the control group that can be termed as a marker of safety.

Furthermore, it can also be said that further modification and isolation of the therapeutic compound of *G. procumbens* may provide us a better effect than metformin, as there is no statistical significance in the difference of the test results between groups 3 and 4. But metformin itself is a single API that was given in doses 100, 250, and 500 mg/kg body weight; on the other hand, the plant extract was given at a dose of 500, 750 and 1,000 mg/kg body weight that contains numerous compounds, and hence, naturally, its antidiabetic effects will be lower than that of metformin.

*G. procumbens* contains significant amounts of bioactive constituents, including flavonoids, phenolic compounds, steroids, alkaloids, terpenoids, and glycosides [50]. Several literature reviews and scientific findings highlighted the importance of flavonoids and phenolic compounds to inhibit the elevation of blood glucose levels. These natural compounds affect plasma glucose levels by inhibiting alpha-amylase and alpha-glucosidase enzymatic activity, hence giving hypoglycemic efficacy. Rutin, nicotiflorin, myricitin, and eriocitrin of flavonoid group and 3,4 dicaffeoylqunic acid, 4,5 dicaffeoylqunic acid, chlorogenic acid, feruloylqunic acid, and glucoronoide from the phenolic group are key compounds and show better binding affinity than acarbose. Thus, these natural compounds refrain from carbohydrate breakdown into glucose in the small intestine and give synergistic effects on acarbose, a glucose-lowering drug.

According to the findings of our study, it was observed that reduction in food intake had no effect on the blood glucose levels of rats. The damaging effects of alloxan-induced cellular atrophy and an initial weight loss in rats treated with alloxan [51]. However, when the rats (both nondiabetic and diabetic) were treated with test extract (*G. procumbens*), their body weights accelerated just like the rats belonging to other groups. So it can be inferred that the mechanism of antidiabetic activity of *G. procumbens* may not be attributed to appetite reduction in rats.

Our study also demonstrated a significant elevation in serum insulin level of rats in groups 6, 7, and 8; whereas no significant change was detected in the insulin level of rats belonging to other groups. Therefore, it can be interpreted that the test extract *(G. procumbens)* may boost insulin secretion by affecting pancreatic beta cells. Furthermore, a significant rise was observed in the hepatic glycogen content of rats from groups 6, 7, and 8, indicating a possible phenomenon of higher insulin levels. Therefore, it can be deduced that the proposed mechanism through which *G. procumbens* mediates its antidiabetic effects is the increase in the release of insulin.

In addition, our investigation indicated that *G. procumbens* can decline the diabetogenic alloxan-induced concentration of free radicals. The anti-oxidant constituents of *G. procumbens* may serve as free-radical scavenging agents, imparting their actions against hydroxyl radical (^•^OH), oxygen radical (O_2_^•−^) as well as lipid peroxidation. This action may also diminish the rate of tissue damage caused by alloxan-induced free radicals.

After a thorough assessment of alpha-glucosidase and natural ligands interaction study, flavonoids and phenolic compounds bind in important amino acid residues of Asp 327, Asp 542, Arg 526, Asp 203, and His 600, which is similar with acarbose binding site [52]. In addition, Trp 59, Thr 163, His 305, Lys 200, Glu 233, and Tyr 151 are important amino acid residues of the binding site for alpha-amylase and acarbose complex [53]. Flavonoids and phenols of *G. procumbens* extracts show better binding affinity, and their interaction study represents a similar binding pattern. There have several scientific studies revealed the antidiabetic property of flavonoids and phenols. Our in silico study also strengthens the previous pharmacological findings on flavonoids and phenols of *G. procumbens*.

## 5. Conclusion

In this experimental study, *Gynura procumbens* leaf extract has shown its extensive use as an herbal treatment for type II diabetes. Our preclinical research in animal models imparted the efficiency of the ethanolic extracts of these leaves with extensive safety study evidence. A dose-dependent improvement of the health status of diseased rats indicated and encouraged the scientists to carry out a further clinical investigation to amplify the effect. An in silico study was conducted to examine an in-depth ligand-target interaction method where natural constituents of *G. procumbens* shared similar interaction sites like acarbose in both alpha-amylase and alpha-glucosidase enzymes. The phytocompounds of this plant gave better binding affinity than acarbose, thus reducing the postprandial hyperglycemia. Overall, it can be concluded that further investigation of *G. procumbens* at the molecular level can be conducted to bring a new dimension to the drug development process.

## Figures and Tables

**Table 1:** Test for peak plasma concentration levels.

Group number	Group status	Treatment specimen	Dose of treatment specimen (mg/kg)	Group abbreviation
1	Alloxan + *G*. *procumbens*	*G. procumbens*	150 mg/kg + 2,000 mg/kg	*g* + *G*_2000_

**Table 2:** Oral glucose tolerance test (OGTT).

Group number	Group status	Treatment specimen	Dose of treatment specimen	Group abbreviation
1	Control	Physiological saline	10 mL/kg	C
2	Glucose control	Glucose	10 gm/kg	g
3	Glucose + metformin	Metformin	10 gm/kg + 100 mg/kg	*g* + *M*_100_
4	Glucose + metformin	Metformin	10 gm/kg + 200 mg/kg	*g* + *M*_200_
5	Glucose + metformin	Metformin	10 gm/kg + 500 mg/kg	*g* + *M*_500_
6	Glucose + *G. procumbens*	*G. procumbens*	10 gm/kg + 500 mg/kg	*g* + *G*_500_
7	Glucose + *G. procumbens*	*G. procumbens*	10 gm/kg + 750 mg/kg	*g* + *G*_750_
8	Glucose + *G. procumbens*	*G. procumbens*	10 gm/kg + 1,000 mg/kg	*g* + *G*_1000_

**Table 3:** Anti-hyperglycemic activity analysis.

Group number	Group status	Treatment specimen	Dose of treatment specimen (mg/kg)	Group abbreviation
1	Control	Physiological saline	10 mL/kg	C
2	Alloxan control	Alloxan	150 mg/kg	A
3	Alloxan + metformin	Alloxan + metformin	150 mg/kg + 100 mg/60 kg	*A* + *M*_100_
4	Alloxan + metformin	Alloxan + metformin	150 mg/kg + 200 mg/60 kg	*A* + *M*_200_
5	Alloxan + metformin	Alloxan + metformin	150 mg/kg + 500 mg/60 kg	*A* + *M*_500_
6	Alloxan + *G. procumbens*	Alloxan + *G. procumbens*	150 mg/kg + 500 mg/kg	*A* + *G*_500_
7	Alloxan + *G. procumbens*	Alloxan + *G. procumbens*	150 mg/kg + 750 mg/kg	*A* + *G*_750_
8	Alloxan + *G. procumbens*	Alloxan + *G. procumbens*	150 mg/kg + 1,000 mg/kg	*A* + *G*_1000_
9	Metformin	Metformin	100 mg/60 kg	*M*_100_
10	Metformin	Metformin	200 mg/60 kg	*M*_200_
11	Metformin	Metformin	500 mg/60 kg	*M*_500_
12	*G. procumbens*	*G. procumbens*	500 mg/kg	*G*_500_
13	*G. procumbens*	*G. procumbens*	750 mg/kg	*G*_750_
14	*G. procumbens*	*G. procumbens*	1,000 mg/kg	*G*_1000_

**Table 4:** Overdose toxicity analysis.

Group number	Group status	Treatment specimen	Dose of the treatment specimen (gm/kg)	Group abbreviation
1	Control	Physiological saline	10 mL/kg	C
2	Alloxan + metformin	Alloxan + metformin	150 mg/kg + 5 gm/60 kg*∗*	A + M
3	Alloxan + *G. procumbens*	*G. procumbens*	150 mg/kg + 25 gm/kg*∗*	A + G
4	Metformin	Metformin	5 gm/60 kg*∗*	M
5	*G. procumbens*	*G. procumbens*	25 gm/kg*∗*	G
*∗*50 times of lower dose = overdose				

**Table 5:** Effect of *G. procumbens* on the activities of glycolytic enzymes in the liver of control and experimental rats.

Group	Hexokinase (*n* moles of glucose-6-phosphate formed/min/mg/protein)	Aldolase (*n* moles of glyceraldehyde formed/min/mg protein)	Phosphoglucoisomerase (*n* moles of fructose formed/min/mg protein)
C	397.26 ± 7.39	156.3 ± 7.13	49.32 ± 5.49
A	103.36 ± 5.86	244.82 ± 30.7	27.32 ± 4.55
A + M_100_	228.62 ± 15.97^∗^	201.9 ± 11.56^∗^	33.28 ± 4.95^∗^
A + M_200_	333.8 ± 11.20^∗^	176.14 ± 8.75^∗^	41.24 ± 1.63^∗^
A + M_500_	401.4 ± 10.05^∗^	155.86 ± 14.2^∗^	49.28 ± 3.40^∗^
A + *G*_500_	213.86 ± 12.20^∗^	210.86 ± 9.94^∗^	30.4 ± 4.21^∗^
A + *G*_750_	325.9 ± 11.05^∗^	192.12 ± 11.25^∗^	38.64 ± 1.50^∗^
A + *G*_1000_	378.56 ± 21.88^∗^	157.64 ± 8.20^∗^	47.38 ± 2.30^∗^
M_100_	391.24 ± 8.56	161.74 ± 6.95	46.74 ± 6.56
M_200_	393.16 ± 8.91	152.58 ± 7.07	50.04 ± 4.43
M_500_	395.64 ± 6.87	151.68 ± 6.34	47.7 ± 3.08
*G*_500_	396.44 ± 8.34	161.92 ± 5.79	48.76 ± 3.98
*G*_750_	388.14 ± 2.53	159.62 ± 11.89	49.06 ± 4.04
*G*_1000_	397.68 ± 7.185	150.08 ± 2.85	50.78 ± 3.64

^∗^ *p* < 0.05 indicates a significant difference from the disease group (C = control group, A = alloxan-treated group, M = metformin, A + M = alloxan + metformin, A + G = alloxan + *Gynura*, and *G* = *G. procumbens*).

**Table 6:** Effect of *G. procumbens* on the activities of glycolytic enzymes in the kidney of control and experimental rats.

Group	Hexokinase (*n* moles of glucose-6-phosphate formed/min/mg/protein)	Aldolase (*n* moles of glyceraldehyde formed/min/mg protein)	Phosphoglucoisomerase (*n* moles of fructose formed/min/mg protein)
C	291.64 ± 10.52	222.7 ± 13.64	31.64 ± 3.67
A	82.7 ± 5.05	286.26 ± 19.86	18.48 ± 2.90
A + M_100_	152.46 ± 6.88^∗^	263.86 ± 11.38^∗^	24.36 ± 2.31^∗^
A + M_200_	234.1 ± 7.3^∗^	243.06 ± 10.98^∗^	27.02 ± 2.99^∗^
A + M_500_	274.94 ± 9.61^∗^	229.54 ± 4.75^∗^	30.3 ± 1.77^∗^
A + *G*_500_	142.32 ± 10.59^∗^	267.5 ± 6.04^∗^	24.98 ± 2.08^∗^
A + *G*_750_	221.92 ± 7.69^∗^	241.94 ± 10.34^∗^	30.16 ± 2.26^∗^
A + *G*_1000_	260.28 ± 8.788^∗^	224.68 ± 7.75^∗^	28.68 ± 2.07^∗^
M_100_	292.56 ± 17.18	225.74 ± 9.55	28.4 ± 3.09
M_200_	290.56 ± 8.18	212.58 ± 2.27	29.62 ± 2.83
M_500_	288.36 ± 9.30	225.38 ± 7.98	30.7 ± 1.93
*G*_500_	291.16 ± 6.76	219.34 ± 7.28	29.98 ± 1.23
*G*_750_	288 ± 13.13	223.74 ± 8.75	32 ± 1.86
*G*_1000_	279.6 ± 12.72	219.42 ± 8.52	32.8 ± 2.07

^∗^ *p* < 0.05 indicates a significant difference from the disease group (C = control group, A = alloxan-treated group, M = metformin, A + M = alloxan + metformin, A + G = alloxan + *Gynura*, and *G* = *G. procumbens*).

**Table 7:** Activities of gluconeogenic enzymes in the liver of control and experimental rats.

Group	Glucose-6-phosphatase (*n* moles of Pi liberated/min/mg protein)	Fructose-1,6-diphosphatase (*n* moles of Pi liberated/min/mg protein)
C	44.968 ± 4.26	33.84 ± 3.51
A	101.94 ± 6.79	347.45 ± 11.54
A + M_100_	79.73 ± 3.27^∗^	62.96 ± 8.18^∗^
A + M_200_	61.98 ± 3.06^∗^	44.18 ± 2.24^∗^
A + M_500_	49.76 ± 3.46^∗^	35.04 ± 1.72^∗^
A + *G*_500_	74.74 ± 8.89^∗^	70.04 ± 7.69^∗^
A + *G*_750_	56.44 ± 3.78^∗^	50.936 ± 5.54^∗^
A + *G*_1000_	46.12 ± 3.072^∗^	40.146 ± 2.40^∗^
M_100_	47.052 ± 3.51	32.458 ± 3.45
M_200_	42.516 ± 2.26	30.62 ± 3.12
M_500_	48.56 ± 2.35	30 ± 0.911
*G*_500_	45.84 ± 2.32	33.52 ± 1.85
*G*_750_	44.652 ± 3.28	31.64 ± 4.05
*G*_1000_	50.22 ± 3.39	31.58 ± 2.03

^∗^ *p* < 0.05 indicates a significant difference from the disease group (C = control group, A = alloxan-treated group, M = metformin, A + M = alloxan + metformin, A + G = alloxan + *Gynura*, and *G* = *G. procumbens*).

**Table 8:** Activities of gluconeogenic enzymes in the kidney of control and experimental rats.

Group	Glucose-6-phosphatase (*n* moles of Pi liberated/min/mg protein)	Fructose-1,6-diphosphatase (*n* moles of Pi liberated/min/mg protein)
C	50.74 ± 4.03	22.1 ± 2.98
A	129.18 ± 5.10	219.88 ± 5.13
A + M_100_	70.2 ± 7.45^∗^	40.32 ± 4.39^∗^
A + M_200_	59.8 ± 5.16^∗^	33.62 ± 4.29^∗^
A + M_500_	48.12 ± 1.71^∗^	26.02 ± 1.60^∗^
A + *G*_500_	74.08 ± 9.51^∗^	45.16 ± 5.74^∗^
A + *G*_750_	62.12 ± 6.09^∗^	38.94 ± 3.38^∗^
A + *G*_1000_	49.14 ± 2.58^∗^	34.24 ± 4.46^∗^
M_100_	52.18 ± 4.56	22.1 ± 2.99
M_200_	50.16 ± 2.9	20.64 ± 2.38
M_500_	49.38 ± 1.94	24.38 ± 2.22
*G*_500_	50.94 ± 3.40	21.04 ± 3.49
*G*_750_	51.04 ± 3.80	19.54 ± 1.84
*G*_1000_	51.34 ± 3.09	21.54 ± 2.45

^∗^ *p* < 0.05 indicates a significant difference from the disease group (C = control group, A = alloxan-treated group, M = metformin, A + M = alloxan + metformin, A + G = alloxan + *Gynura*, and *G* = *G. procumbens*).

**Table 9:** Binding affinity of natural ligands of *G. procumbens* with the crystal target alpha-amylase.

Compound name	Binding affinity	Class of compounds
Daucosterol	−10.5	Steroid
Glucopyranosyl stigmasterol	−9.9	Steroid
1,5-Dicaffeoylquinic acid	−9.6	Phenol
3,5-Dicaffeoylquinic acid	−9.6	Phenol
Luteolin	−9.5	Flavonoid
Beta-stigmasterol	−9.5	Steroid
3,4-Dicaffeoylquinic acid	−9.4	Phenol
Eriocitrin	−9.4	Flavonoid
Apigenin	−9.3	Flavonoid
Rutin	−9.3	Flavonoid
Quercetin	−9.2	Flavonoid
Homoorientin	−9.1	Flavonoid
Nicotiflorin	−9.1	Flavonoid
5*α*-Stigmastan-3-one	−9.1	Steroid
4,5-Dicaffeoylquinic acid	−9.1	Phenol
Myricetin	−9	Flavonoid
1,4-Dicaffeoylquinic acid	−8.9	Phenol
Kaempferol	−8.8	Flavonoid
Glucuronide	−8.8	Phenol
Negletein	−8.7	Flavonoid
Senecivernine	−8.6	Alkaloid
Seneciphylline	−8.5	Alkaloid
Astragalin	−8.4	Flavonoid
Senkirkine	−8.3	Alkaloid
Senecionine-N-oxide	−8.3	Alkaloid
Erucifoline-N-oxide	−8.1	Alkaloid
Retrorsine	−8.1	Alkaloid
Neochlorogenic acid	−8	Phenolic
Seneciphylline-N-oxide	−8	Alkaloid
Chlorogenic acid	−7.9	Phenol
Feruloylquinic acid	−7.7	Phenol
Acarbose	−7.6	Reference

**Table 10:** Binding affinity of natural ligands of *G. procumbens* with the crystal target of N-glucosidase.

Compound name	Binding affinity	Class of compound
Nicotiflorin	−9.3	Flavonoid
Rutin	−9.2	Flavonoid
Beta-stigmasterol	−9.1	Steroid
5*α*-Stigmastan-3-one	−8.8	Steroid
3,4-Dicaffeoylquinic acid	−8.7	Phenol
4,5-Dicaffeoylquinic acid	−8.7	Phenol
Eriocitrin	−8.7	Flavonoid
Glucopyranosyl stigmasterol	−8.5	Steroid
1,5-Dicaffeoylquinic acid	−8.5	Phenol
1,4-Dicaffeoylquinic acid	−8.3	Phenol
3,5-Dicaffeoylquinic acid	−8	Phenol
Astragalin	−7.7	Flavonoid
Luteolin	−7.6	Flavonoid
Myricetin	−7.6	Flavonoid
Feruloylquinic acid	−7.5	Phenol
Chlorogenic acid	−7.5	Phenol
Negletein	−7.5	Flavonoid
Quercetin	−7.5	Flavonoid
Apigenin	−7.5	Flavonoid
Neochlorogenic acid	−7.5	Phenol
Glucuronide	−7.5	Phenol
Homoorientin	−7.4	Flavonoid
Kaempferol	−7.4	Flavonoid
Caffeoylglucaric acid	−7.3	Phenol
Hydroxytyrosol glucoside	−7.2	Phenol
Daucosterol	−7.2	Steroid
Senecionine-N-oxide	−7.1	Alkaloids
Senkirkine	−7	Alkaloids
Seneciphylline-N-oxide	−6.9	Alkaloids
Acarbose	−6.7	Reference

## Data Availability

All data are included within the paper.
